# Social conditions impact functional outcome in patients with hand osteoarthritis: the low-income hand osteoarthritis (LIHOA) cohort

**DOI:** 10.1186/s42358-024-00420-9

**Published:** 2024-10-04

**Authors:** Francisco Vileimar Andrade de Azevedo, João Pedro Sobreira Borges, Antonio Matos de Souza Filho, José Carlos Godeiro Costa Junior, Cláudio Régis Sampaio Silveira, Francisco Airton Castro Rocha

**Affiliations:** 1 Postgraduate Program in Medical Sciences, Federal University of Ceará, Fortaleza, Brazil; 2 Department of Internal Medicine, Faculty of Medicine, Universidade Federal do Ceará, Fortaleza, CE Brazil; 3 Instituto São Carlos de Ensino e Pesquisa\São Carlos Imagem, Fortaleza, Ceará Brazil; 4 Instituto de Biomedicina, Laboratório de Investigação em Osteoartropatias, Rua Coronel Nunes de Melo, 1315 -1º. Andar, Rodolfo Teofilo, Fortaleza, CE CEP: 60430-270 Brazil

**Keywords:** Osteoarthritis, Hand, Social condition, Functional outcome, Pain, Income

## Abstract

**Background:**

Hand osteoarthritis (HOA) is a highly prevalent disease that may be impacted by social inequalities. Few studies in HOA are from underdeveloped regions. We intend to contribute to fill this gap presenting clinical characteristics of our low-income HOA cohort (LIHOA).

**Methods:**

Data from 119 patients with a HOA diagnosis fulfilling ACR criteria seen between August 2019 and May 2023 in Fortaleza/Brazil. Evaluations included pain (VAS, visual analogue scale), X-ray (KL, Kellgren-Lawrence), grip and pinch strength (KgF), Cochin hand functional scale (CHFS), FIHOA, and SF-12 scores. Social data included monthly (<1, 1≥/<3, ≥3 MW) minimum wage earnings, occupation, and literacy [</≥ 9 school-years (SY)].

**Results:**

107 out of the 119 patients were included. Mean age was 61.9 (±10.3) years with 94 (92%) women. Systemic arterial hypertension (48%), metabolic syndrome (42.8%), dyslipidemia (28.4%), and obesity (25%) were the most common comorbidities. Mean disease duration was 7.5 ± 7.1 years. Median VAS values at rest and activity were 3 (3–5) and 8 (5–9), respectively (*p* < 0.001). Fifty-seven (56.4%) patients had ≥4 symptomatic joints with a median of 4 (2–8) painful joints at activity. The 2nd distal interphalangeal (IF), joint was the most symptomatic (21; 23.3%) and most had >4 IF nodes. OA in other joints: 37 (36.2%) spine, 28 (29.4%) knee, 21 (20.5%) bunions. Functional impairment was mild [8 (5–14) median FIHOA]. Median serum CRP was 0.2 mg/dL (0.1–0.4) with 14 (20%) patients above reference value. Mean total KL score was 27.6 ± 13.6 with 21 (23%), 38 (41.7%), and 33 (36.2%) KL2, KL3, and KL4, respectively; 51 (54.8%) and 42 (45.2%) patients declared </≥3 MW earnings, respectively. Most declared >9SY including 37.2% with a university degree. Individuals earning <3 MW had lower pinch (*p* < 0.004) and grip strength (*p* < 0.01), and higher FIHOA scores (*p* < 0.007), as compared to ≥3 MW earning group. Literacy or occupation did not impact outcome. SYSADOA were used by 13 (12.7%), 6 used oral and 3 topical anti-inflammatory drugs and 2 used 5 mg/d prednisone.

**Conclusion:**

Clinical characteristics in our LIHOA cohort mirror those reported in affluent regions. Socioeconomic disparities influenced functional outcome in LIHOA cohort.

## Introduction

Hand osteoarthritis (HOA) is a highly prevalent musculoskeletal disorder, often involving multiple joints of the hand with various overlapping phenotypes that include nodal HOA, osteoarthritis of the base of the thumb and erosive HOA [1–3]. Osteoarthritis of the hands can be defined by the American College of Rheumatology (ACR) clinical criteria, by structural alterations established by radiology, namely radiographic HOA, as well as radiographic alterations accompanied by typical symptoms, termed symptomatic HOA [4, 5]. Previous data indicate uncoupling of the prevalence of symptomatic and radiographic HOA. Indeed, while the prevalence of radiographic HOA ranges from 21 to 92% that of symptomatic HOA is much lower, ranging from 3 to 16% in different populations [6–11]. Nodes in the interphalangeal (IP) joints are a hallmark of nodal HOA, being associated with underlying radiographic alterations caused by this disease [12, 13]. Radiographically, HOA may manifest in three different patterns: finger OA, thumb OA, and combined thumb and finger OA [2, 5, 14]. The heterogeneity of the clinical picture of HOA, with diverse phenotypes, has been considered an issue when trying to evaluate treatment modalities in these patients [2, 5, 15]. Social inequalities have been shown to impact disease presentation worldwide [16]. However, data on prevalence and clinical presentation of HOA to date were mostly generated among individuals living in affluent regions [2, 3, 11, 17]. Our study intends to diminish this gap presenting demographic, clinical, laboratory, and radiographic data of a non-Caucasian, low-income cohort with symptomatic nodal HOA (LIHOA). We also determined whether socioeconomic issues impact the severity of clinical and/or radiological HOA characteristics in our cohort.

## Methods

### Study design and setting

Low Income HOA (LIHOA) is a single-center cross-sectional study of patients with symptomatic radiographic HOA consecutively recruited by rheumatologists working in both public or private settings in Fortaleza, CE, Brazil (3°46′S/38°34′W) from August 2019 to May 2023. Patients were interviewed to collect demographic and clinical data, followed by a thorough clinical examination including careful assessment of their hands. All participants gave written informed consent before any procedure. This study complied with current guidelines for good clinical practice and was approved by the ethics committee of the Universidade Federal do Ceará (CAAE: 02879318.8.1001.5054).

#### Inclusion criteria

Individuals ≥40 years-old with nodal symptomatic HOA according to ACR criteria [4] with ≥2 symptomatic joints among proximal/distal IP joints or 1st IP joint and radiographic Kellgren–Lawrence (KL) stage ≥2. Symptomatic HOA was defined by the presence of pain plus stiffness or aching in an IP joint on at least half of the days in the last 4 weeks. Although patients with erosive HOA have been included, this characteristic was not specifically addressed in the present study.

#### Exclusion criteria

Diagnosis of inflammatory arthritis particularly rheumatoid and/or psoriatic arthritis; gout or calcium dihydrate pyrophosphate disease; infections or acute trauma to the hands; hereditary hemochromatosis; carpal tunnel syndrome, De Quervain’s tendinopathy, Dupuytren’s disease, diabetic neuropathy, thoracic outlet syndrome, previous upper limb surgery.

### Socioeconomic data

Income evaluation considered monthly family income using March 2022 as reference for conversion of Brazilian R$ to US$ currency (1US$ = 4.96R$), based on monthly minimum wage (MMW), as follows: <1, 1≥/<3, ≥3 MW, which corresponded roughly to <300.00 US$, 300.00 ≥/< 900.00 US$, and ≥900.00 US$. Families earning <3 MW were considered as low-income, according to official data [18]. Literacy was classified as </≥ 9 school-years (SY) or university degree, considering individuals with <9 SY as low literacy, also according to official data [19]. Current or previous occupations were arbitrarily classified as either blue/white collar jobs using the Brazilian classification of occupations [20].

### Clinical and laboratory data

A case report form was used to register all clinical data including disease duration, presence of continuous chronic pain in hand joints, and the number of pain flares occurring over a six-month period prior to study entry. Pain in the hand joints (at rest, activity and upon palpation), presence of soft tissue swelling (i.e., capsule-synovial enlargement), most painful joint in both hands, as well as the number of distal and proximal IP nodes (Heberden’s and Bouchard’s nodes, respectively) were registered. Pinch (pulp pinch) and grip strength of the most painful finger and the most symptomatic hand were measured using a Saehan^®^ hydraulic pinch gauge and a hydraulic hand dynamometer, respectively, registering the best result of 3 tests. Patient reported outcomes were assessed using fulfilment of the Functional Index for HOA (FIHOA), Cochin hand functional disability scale (CHFS), the 12-item Short Form Health Survey (SF-12) questionnaires as well as patient self-assessed level of pain in the most symptomatic joint at rest and activity, using a 0–10 cm visual analogue scale (VAS) [21–23]. Concomitant OA in joints other than the hands (shoulders, cervical and lumbar spine, hips and knees) was registered based on available clinical and imaging data. Knee OA diagnosis was made using the ACR clinical criteria and hallux valgus (bunion) secondary to OA was determined based on clinical history to exclude other causes, physical examination, and presence of a hallux valgus angle ≥ 20° [24, 25]. Clinical examination was done by two senior board-certified rheumatologists (FVAA, FACR). Comorbidities including hypertension, diabetes, and obesity (Body mass index, BMI ≥ 30), and metabolic syndrome diagnosed using the American Association of Clinical Endocrinologists (AACE) criteria were registered [26]. A fasting blood sample was collected for routine workup, including the determination of erythrocyte sedimentation rate and serum C-reactive protein levels (CRP). Ongoing pharmacological treatments for OA were also recorded, including nonsteroidal antiinflammatory drugs (NSAIDs), opioids, and symptomatic slow acting drugs for OA (SYSADOA), encompassing nutraceuticals and phytocompounds.

### Imaging data

Postero-anterior views of hand radiographs were scored using the Kellgren-Lawrence (K-L) scale by two senior board-certified radiologists blinded to clinical data (CRSO, JCGCJr) registering the maximum score among the 4 distal and proximal IP joints (DIP and PIP, respectively), and the IP of the thumb [27].

### Statistical analysis

Characteristics of the patients were expressed as frequencies and percentages for categorical variables and as means ± standard deviation (SD) or medians (range) and interquartile ranges (IQRs) for continuous variables, as appropriate. Comparisons were made using Student’s “t” test or Mann-Whitney, as appropriate. The level of significance was set at 0.05. Analysis of data were done using SAS 9.4 M7, SAS Inc.

## Results

### Clinical characteristics

**Fig. 1:**
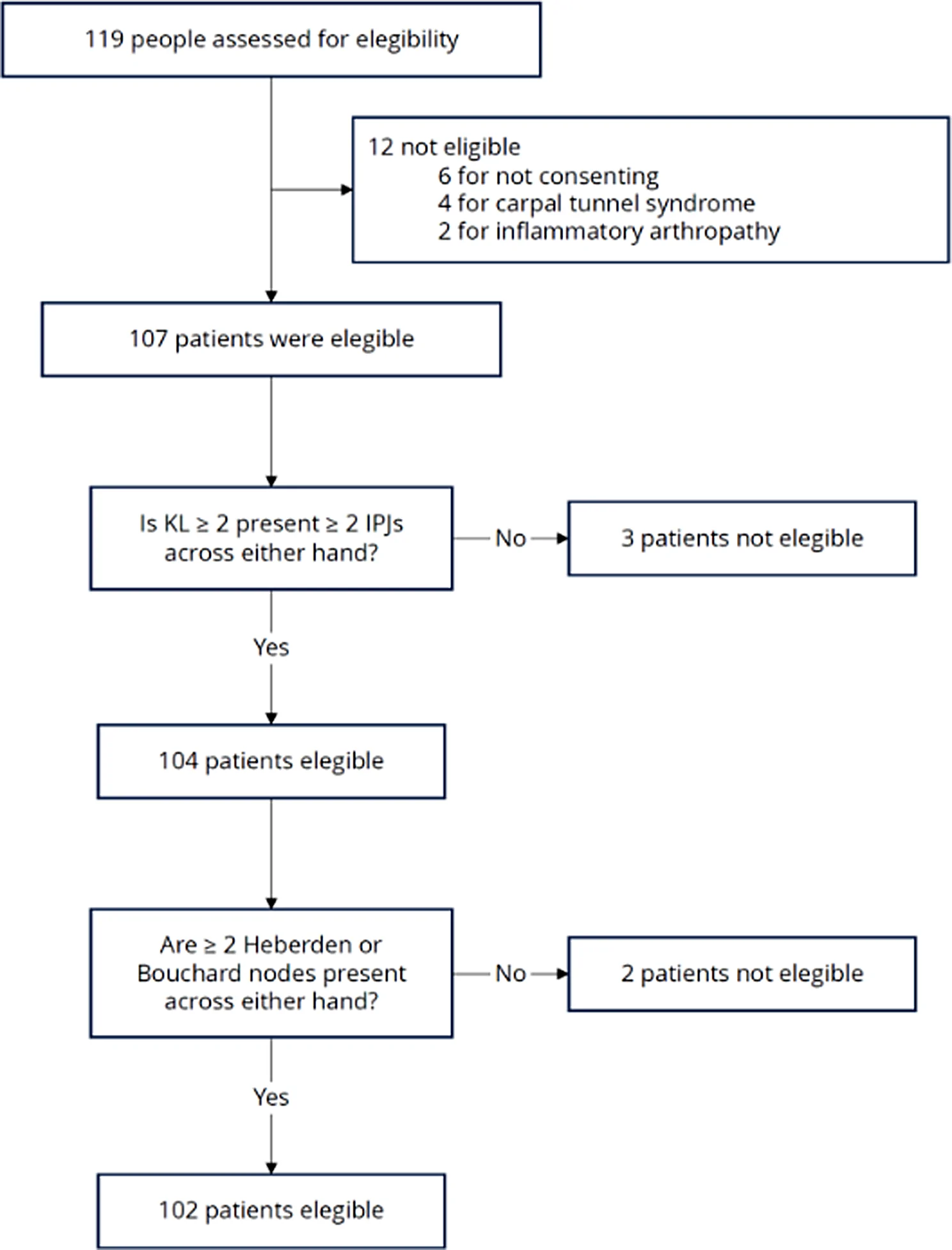
Flow chart of individuals included in the LIHOA cohort

Figure 1 illustrates a flow chart of the 119 recruited patients. Initially, 12 patients were excluded, as follows: 6 met exclusion criteria, 6 declined to participate, 3 did not meet radiologic criteria and 2 had no IF nodes. Mean age was 61.9 (±10.3) years-old with 53.9% participants over 60 years-old, the oldest being 89 years-old. Women were highly predominant (94; 92%). Systemic arterial hypertension (48%), metabolic syndrome (42.8%) and dyslipidemia (28.4%) were the most common comorbidities, whereas obesity was present in 23 (25%) individuals with 3 presenting 41, 41, and 42 as the higher BMI values (Table 1). Mean disease duration was 7.5 ± 7.1 years. Pain on activity was significantly higher than at rest (*p* < 0.001) with patients being mildly to moderately symptomatic on joint assessment at rest and activity, with 3 (3–5) and 8 (5–9) median VAS values, respectively. Fifty-seven (56.4%) patients had ≥ 4 symptomatic joints with a median of 4 (2–8) painful joints at activity. The 2nd distal IF joint was the most symptomatic (21; 23.3%), followed by the 3rd distal IF (19; 21.1%). Most patients had over 4 IF nodes and the majority had OA in joints other than the hands, as follows: 37 (36.2%) in the lumbar and/or cervical spine followed by 28 (29.4%) with knee OA and 21 (20.5%) hallux valgus. Overall functional impairment was considered mild based on a median 8 (5–14) FIHOA value (Table 2).

**Table 1:** Baseline characteristics

	Number data available	
Gender	102	
Female		94 (92)
Male		8 (7.8)
Age (years)	102	61.9 ± 10.3
Current professional situation	95	
Active		61 (64.3)
Retired		34 (35.7)
Main occupation (past or current)	98	
Blue Collar		41 (41.8)
White Collar		57 (58.1)
Literacy	97	
≤8 years		15 (15.4)
>8 years		82 (81.6)
Monthly family income	93	
<1 MW		17 (18.3)
1≤ MW <3		34 (36.5)
≥3 MW		42 (45.2)
BMI (kg/m²)	92	27.3 ± 1
Obese (≥30)		23 (25)
Comorbidities	102	
Hypertension		49 (48)
Metabolic syndrome		36 (42.8)
Dyslipidemia		29 (28.4)
Osteoporosis		15 (14.7)
Generalized anxiety disorder		12 (11.7)
Thyroid disease		13 (12.7)
Diabetes mellitus		11 (10.7)
Migraine		9 (8.8)
Depression		6 (5.8)
Cardiovascular disease		7 (6.8)

Data are mean (SD), or N(%); *BMI* body mass index

**Table 2:** LIHOA cohort—clinical variables at baseline

	Number data available	
Symptom duration (years)	95	7.5 ± 7.1
Hand pain intensity at rest	95	3 (3–5)
Hand pain intensity at activity	95	8 (5–9)
Hand pain profile	91	
Continuous pain		55 (66.5)
Evolution by flares		36 (39.5)
Total number of IF nodes	96	8 (6–10)
≤3 nodes		11 (11.4)
>3 nodes		85 (93.7)
Heberden’s nodes		7 (5–8)
Bouchard’s nodes		1 (0–2)
Number of painful joints at activity	101	4 (2–8)
Number of painful joints at palpation	101	4 (2–8)
Number of joint soft tissue swelling	101	4 (2–8)
Presence of OA at other sites	102	70 (68.6)
Spine		37 (36.2)
Knee OA		28 (29.4)
Rhizarthrosis		19 (18.6)
Shoulder		9 (8.8)
Hip		2 (1.9)
Halux valgus (Bunion)		21 (20.5)
Grip strength (kg)	87	14.9 ± 6.6
Pinch strength (kg)	87	1.9 ± 1.2
FIHOA	101	8 (5–14)
Cochin	102	19 (6–29)
SF12	89	32 (31–34)
Physical component		36.6 (9.1)
Mental component		50.8 (10.1)

Data represent mean (SD), medians (IQR), or N (%); *OA* osteoarthritis, *IFJ* interphalangeal, *FIHOA* Functional index for hand osteoarthritis, *SF12* 12-item Short Form

### Laboratory and imaging data

Laboratory and imaging data are reported in Table 3 showing 0.2 mg/dL (0.1–0.4) median serum CRP level with 14 (20%) patients displaying CRP levels above reference value (0.5 mg/dL). The mean total KL score of the 95 analysed radiographs was 27.6 ± 13.6 with 21 (23%), 38 (41.7%), and 33 (36.2%) patients classified as KL2, KL3, and KL4, respectively.

**Table 3:** LIHOA cohort—laboratory data at baseline

	Data available	*N* (%) or median
Serum CRP (mg/dL)	69	0.2 (0.1–0.4)
Number of patients < 0.5 mg/dL		55 (80)
Number of patients ≥ 0.5 mg/dL		14 (20)
Total cholesterol (mg/dL)	69	0.2 (0.1–0.4)
LDL cholesterol (mg/dL)	67	127 (105–138)
HDL cholesterol(mg/dL)	68	53 (46.7–62.2)
Triglyceride (mg/dL)	70	122 (91.2–149)
Fasting glycemia (mg/dL)	60	94 (90–99)
HbA1c (%)	60	5.6 (5.3–5.9)
Hemoglobin (g/dL)	84	13.2 (12.8–13.7)

Data represent median (IQR) or N(%); *CRP* C-reactive protein, *HbA1c* glycated hemoglobin

### Socioeconomic data

Ninety-three individuals agreed to inform family income, with 51 (54.8%) and 42 (45.2%) declaring earnings </≥3 MW, respectively, with a slight majority being classified as having white-collar occupations. Most patients declared >9SY of formal education, with 37.2% having a university degree. Individuals earning <3 MW had lower pinch (*p* = 0.004) and grip strength (*p* = 0.01) scores as well as higher FIHOA scores (*p* = 0.007), as compared to those declaring ≥3 MW earnings (Table 4). There were no significant differences regardless of literacy or occupation (data not shown).

**Table 4:** Impact of family income in hand osteoarthritis

	Low	High	Mean difference (95% CI)	*P* value
N° painful joints at activity	5.9	4.7	−1.14 (−3.2, 0,91)	0.273
Nº painful joints at palpation	8.1	6.5	−0.83 (−3.76, 0.68)	0.173
Nº joints soft tissue swelling	6.0	5.2	−1.53 (−2.6, 0.91)	0.346
Hand pain at rest (VAS)	4.1	3.5	−0.59 (−1.55, 0.35)	0.217
Hand pain at activity (VAS)	7.2	7.1	−0.09 (−1.05, 0.86)	0.848
Total number of IF nodes	8.28	7.78	−0.50 (−2.04, 1.03)	0.518
Nº other sites with osteoarthritis	1.1	1.0	−0.10 (−0.53, 0.31)	0.608
Pinch strengh (kg)	1.4	2.2	0.81 (0.26, 1.35)	0.004
Grip strengh (kg)	13.2	16.8	3.60 (0.66, 6.54)	0.017
Cochin	21.5	17.1	−4.37 (−10.6, 1.92)	0.171
FIHOA	11.1	7.4	−3.69 (−6.36, −1.09)	0.007
SF-12	32.1	32.6	0.52 (−0.70, 1.76)	0.398
KL score (total)	32.2	27.5	−4.66 (−14, 4.72)	0.319
Sérum CRP (mg/dl)	0.44	0.24	−0.19 (−0.46, 0.07)	0.5

Patients with Hand osteoarthritis were classified as having Low or High monthly family income. *CRP* serum C-reactive protein, *FIHOA* Functional index for hand osteoarthritis, *KL* Kellgren-Lawrence, *SF12* 12-item Short Form questionnaire, *VAS* visual analogue scale

### Treatments

Almost 80% of the patients were not taking any specific medications to treat their HOA; SYSADOA prescribed by physicians were used by 13 (12.7%) individuals, comprising 6 (5.8%), 5 (5%), and 2 (1.9%) using hydrolyzed collagen, glucosamine sulfate/chondroitin sulfate (5), and *Harpagophytum procumbens* (1), respectively; oral and topical NSAIDs were being used by 6 (6%) and 3 (3%) patients, respectively; 2 (2%) patients were using 5 mg/d oral prednisone. No patient had undergone or was planning to undergo surgery for HOA.

## Discussion

Our work describes the clinical characteristics of individuals of a symptomatic low-income HOA (LIHOA) non-caucasian cohort. In addition to reporting data from underrepresented populations, we also aimed to determine whether social disparities in patients living in the same region have a clinical impact in HOA. Self-declared monthly family income, occupation, and level of literacy were used as surrogates for classifying patients as pertaining to more or less favoured social environments. In an attempt to collect data from different social *strata* in our region, we invited rheumatologists working in both public and private practice to indicate patients to be included in our sample. It turns out that this strategy led us to include over 80% of individuals declaring ≥ 300.00 US$ monthly family earnings, including a slight majority earning > 900.00 US$. Although these higher values are far below earnings declared in affluent populations [28], individuals of the LIHOA cohort do not represent the majority of the population living in our region. Indeed, using official data, our mean 2022 Growth domestic product (GDP) *per capta* was R$ 1023.00 (Brazilian currency), which is roughly <200US$ [28]. However, our strategy allowed us to compare the clinical picture among individuals of different socioeconomic status living in the same region. This can also be illustrated by the fact that most patients declared more than 9SY of formal education with a third having a university degree. By comparison, official data show that only 19.2% of the population living in our region have a university degree [29]. We also have recently published data on juvenile idiopathic arthritis and orthopaedic surgeries, in which patients were recruited consecutively, showing a high prevalence of low-income patients thus reflecting our official data [30, 31]. We ended up finding that individuals classified as low-income had more impact in hand function, showcasing lower pinch and grip strength values and higher FIHOA scores, whereas pain levels did not differ among both groups. Interestingly, neither having a blue or white collar occupation nor the level of literacy influenced symptom severity and/or function in this population. We believe these are the first data showing that income, but not occupation, in patients sharing the same environment, has a significant impact in the function of patients with HOA.

Similar to other cohorts [3, 11, 17], the vast majority of our patients were female, middle-aged, with a long-term HOA disease and moderate to severe pain at movement, with mild to virtually no pain at rest. Comorbidities also mirrored data from other cohorts [17] with hypertension, metabolic syndrome, and dyslipidemia being the most common, followed by osteoporosis, thyroid disease, mood disorders, and diabetes. Interestingly, most patients had concomitant OA in joints other than the hands, most commonly in the spine, followed by the knee. Indeed, in the DIGICOD study, reporting a cohort of HOA patients in France, 25.8% of the patients fulfilled criteria for knee OA, which is similar to the 29.4% frequency of knee OA in our study [17].

It is also worth noting that 20.5% of the patients had bunions that were attributed to OA, which was also as common as the 18.6% of patients that had rizarthrosis. We are not aware of previous studies in HOA patients that assessed the prevalence of concomitant OA in other joints. However, in the DIGICOD cohort, presence of spinal (cervical and/or lumbar) and knee pain, which could be linked to OA, were common musculoskeletal complaints [17].

Functional impairment can be considered mild in our study, given the median values of 8 and 19 in the FIHOA and Cochin questionnaires, respectively. This is very similar to the baseline 10 score in the FIHOA questionnaire reported in the EHOA trial evaluating etanercept as a treatment for hand osteoarthritis [32]. Interestingly, patients in the recently developed DIGICOD cohort had higher FIHOA scores, meaning greater functional impairment, as compared to our results. Given that we did not specifically determined the percentage of patients with erosive HOA in our sample, we can only speculate whether the inclusion of 45.8% individuals with erosive HOA led to higher FIHOA scores in the DIGICOD cohort as compared to our data [17]. Values obtained with the SF-12 questionnaire also mirrored data reported in the HERO study, showcasing a substantial deficit in both mental and physical domains, as compared to healthy individuals [33]. Similar results were reported in the EHOA clinical trial, which included patients with erosive osteoarthritis, showing baseline Physical Component SF-36 values of 42.9, which are lower than the 50 score threshold considered as normal [34, 35]. These data are intriguing, as only 5.8% of our patients declared having a diagnosis of depression and only 11.7% reported having a generalized anxiety disorder. In conjunction, these results suggest a significant mental burden in patients with HOA. Whether this can be specifically attributed to the HOA itself should be further explored.

The low-income subgroup of our cohort presented worse hand function and lower performance in pinch and grip strengths. Those with lower income were also more frequently classified as having blue-collar jobs, with 59% vs. only 21% in the higher-income group. However, there was no statistically significant difference regarding pain, function, imaging or patient reported outcome parameters regardless of patients being classified of having a blue or white collar occupation. We should also consider that this classification does not necessarily reflect other daily-life activities that may impact pain or function in HOA. Moreover, the low number of individuals in each subgroup might have also impacted the analysis. A large majority of patients were not using medications to treat their HOA. This might illustrate a major unmet need regarding pharmacological treatments for pain relief in this disease.

Our study has several limitations, which includes being a single-center study and the cross-sectional analysis. The fact that it was initiated shortly prior to the COVID-19 pandemic severely affected the inclusion of patients. We also did not focus the erosion pattern, as we intended to recruit symptomatic HOA patients. Also, our strategy to enhance the number of individuals of higher income, aiming to find differences regarding social disparities in the same sample, has biased the recruitment by reducing the number of low-income individuals included, which does not reflect the majority of our population. Another limitation is the fact that we did not adjust the statistical evaluation for potential confounders.

## Conclusion

In summary, this is the first study to collect socioeconomic data using a low-income non-Caucasian cohort of HOA patients. Our results are similar to those obtained in affluent regions while indicating that social disparities affect functionality in HOA patients.

## Data Availability

All data generated or analysed during this study are included in this published article.

## Abbreviations

ACR: American College of Rheumatology
BMI: Body mass index
CHFS: Cochin hand functional disability scale
CRP: C-reactive protein levels
DIP: Distal interphalangeal
EHOA: Erosive hand osteoarthritis
FIHOA: Functional index for Hand Osteoarthritis
HOA: Hand osteoarthritis
IP: Interphalangeal
KL: Kellgren–Lawrence
LIHOA: Low income hand osteoarthritis
MW: Minimum wage
NSAIDs: Nonsteroidal antiinflammatory drugs
PIP: Proximal interphalangeal
SY: School-years
SF-12: Short Form Health Survey 12-item
SYSADOA: Symptomatic slow acting drugs for Osteoarthritis
VAS: Visual analogue scale
